# Editorial: Emerging and re-emerging viral infections: epidemiology, pathogenesis and new methods for control and prevention

**DOI:** 10.3389/fpubh.2024.1528163

**Published:** 2025-01-06

**Authors:** Jawhar Gharbi, Giovanni Rezza, Manel Ben M'hadheb

**Affiliations:** ^1^Department of Biological Sciences, College of Science, King Faisal University, Al-Ahsa, Saudi Arabia; ^2^Higher Institute of Health, Department of Infectious Diseases, Ministry of Health, Rome, Italy

**Keywords:** emerging and re-emerging viruses, epidemiology, pathogenesis and virulence, genetic evolution, vaccines, diagnosis, antiviral drugs

Viruses are always transmitted directly and/or indirectly from human to human and from animal to human. In host cells, virus replication frequently results in an accumulation of mutations, reassortments, homologous, and heterologous recombinations, contributing to their rapid adaptation to environmental changes, often raising to the emergence of new virus variants or species. These viral characteristics, in addition to spillover events, have resulted recently in an increasing number of outbreaks, epidemics, and pandemics.

Emerging viruses are a very broad category that includes not only newly discovered viruses but also re-emerging variants of known viruses. In the last 20 years, an alarming number of infectious viruses have emerged or re-emerged, presenting great threats to global public health. Ebola, Marburg, and Crimean Congo hemorrhagic fevers, Lassa fever, Dengue fever, Yellow fever (YFV), West Nile fever (WNV), Rift Valley fever (RVF), Nipah and Hendra viruses, Zika virus, Poxvirus, Hepatitis E Virus (HEV), Bunyavirus and Chikungunya vector-borne viruses, severe acute respiratory syndrome (SARS), Middle East respiratory syndrome (MERS-CoV), and the most recent coronavirus disease 2019 (COVID-19) are examples of zoonoses that have spread throughout the globe with such a significant impact on public health (1, 2). These viruses continue to cause mass disruption by creating constant threat to public health. The scientific community are always called for a rapid intervention in diagnosing, preventing and treating emerging infections (1, 3). Vaccination is probably the most effective tool in helping the immune system to activate protective responses against pathogens, reducing morbidity and mortality, as proven by historical records and the most recently pandemic situation with the emergence of SARS-CoV-2 virus. Under health emergency conditions, new and development of alternative approaches in antiviral drug and vaccine design are imperative for a rapid and massive vaccination coverage, to manage a disease outbreak and curtail epidemic spread. The emergence and re-emergence of novel pathogens challenging the public health in regards to the development of new diagnostic methods, therapeutics and prevention strategies and maintaining an efficient epidemiological surveillance.

The world remains burdened by high morbidity and mortality diseases and, as exemplified by the recent devastating pandemic of SARS-CoV-2, and new emerging or re-emerging pathogens are likely to spread in the future. In this line, this Research Topic collection, “*Emerging and re-emerging viral infections: epidemiology, pathogenesis and new methods for control and prevention*” provides for researchers a wide range of selected articles presenting the last results of the epidemiology and pathogenesis of a numerous of emerging or re-emerging virus pathogens. It offers an update on current antiviral strategies to manage and control these emerging and re-emerging viruses, including vaccines and antiviral drug discovery.

During the management of this Research Topic collection, we received a considerable number of articles with a good scientific level and sound. We selected the most interesting articles belonging to different categories of papers: One as Case report, 1 as Clinical trial, 1 as Methods, 4 as Opinion, 3 as Review, and finally 20 articles as Original research. All article categories covers the important topics of the collections (Genetic evolution and diversity, diagnosis, antiviral drugs, vaccines…). An important number of articles described the recent pandemic of COVID-19 and presented interesting results regarding the study of the pandemic, the emerged associated virus SARS-CoV-2 and the efficiency of used vaccines. The effectiveness of vaccines categories used during the pandemic of COVID-19 were studied and compared within different populations. Du et al., studied the global trends in COVID-19 incidence and case fatality rates as a retrospective study. They concluded that COVID-19 rates varied significantly by continent and income level. Europe and the Americas faced surges in infections and low case fatality rates. Whereas, Africa experienced low infection rates and higher case fatality rates, with lower-and middle-income nations exceeding case fatality rates in high-income countries over time. The impact of the COVID-19 infection and prevention on lower respiratory tract infection pathogenesis and outcomes was then analyzed by Feng et al.. Study demonstrated that the effects of COVID-19 pandemic on the epidemiological characteristics of respiratory pathogens, which will be beneficial for improving early preventive measures and that intervention encompassing pulmonary and functional rehabilitation exercises, are recommended to improve physical fitness and pulmonary function post-COVID-19.

The genetic evolution and diversity of variants detected of the emerging virus associated to the recent pandemic SARS-CoV-2 were also presented in the articles of Moniz et al., Liu et al., Falasca et al., and Zhu et al., which are involved in this Research Topic. Indeed, Moniz et al., reviewed the individual risk factors associated with SARS-CoV-2 Alpha variant infection in high-income countries indicating the multitude risks factors associated to several pathologies including diabetes, cancer, asthma and cardiovascular diseases. Whereas, Liu et al., suggested that Asymptomatic and symptomatic patients infected with Omicron variant had pulmonary involvements which were not uncommon. Potential risk factors for age stratification, and pulmonary diseases can help clinicians to identify obvious pulmonary involvements in asymptomatic and mildly symptomatic patients infected with Omicron. Falasca et al., presented results of the antimicrobial resistance study during the COVID-19 pandemic and before the pandemic. Authors demonstrated how the COVID-19 pandemic changed the prevalence of sepsis in patients in intensive care. They revealed that the risk factors associated with mortality were APACHE and SOFA scores, age, and, above all, the presence of ESBL-producing bacteria. Despite this, during the pandemic phase, they have observed a significant reduction in the emergence of resistant germs compared to the pre-pandemic phase (Falasca et al.). Finally, Zhu et al., examined the epidemiological, genomic, and evolutionary characteristics of the SARS-CoV-2 genomes from China. They analyzed nearly 20,000 genomes belonging to 17 lineages, predominantly including BF.7.14, DY.2, DY.4, and BA.5.2.48 variants. They identified 43 core mutations in the S gene and 47 core mutations in the ORF1ab gene responsible of the evolution of the virus genome. Their findings provide insights into the genomic characteristics of SARS-CoV-2 genomes in China following the relaxation of the “dynamic zero-COVID” policy and emphasize the importance of ongoing genomic monitoring (Zhu et al.).

Other articles from this Research Topic cover different topics regarding various types of emerging or re-emerging viruses such as arboviruses (Dengue, Murray Valley Encephalitis, Nipah…), Monkeypox virus, Hepatitis viruses, and Human immunodeficiency virus. The review of Braddick et al., supported the integrated public health in response to the outbreaks of mosquito-borne flavivirus, the Murray Valley encephalitis virus (MVEV) infection during mosquito seasons in Victoria, Australia. They concluded that the expanded, climate-informed vector surveillance system detected MVEV in mosquitoes in advance of human cases, acting as an effective early warning system. This informed a one-health oriented public health response including enhanced human, vector and animal surveillance, integrated mosquito management, and health promotion (Braddick et al.). The serum of Hepatits B Virus (HBV) was supposed by Yu et al., to be a promising biomarker for blood product safety screening and enhanced diagnostic efficiency in chronic HBV infection. They suggest that the incorporation of serum HBV RNA detection into clinical practice and the implementation of blood safety precautions helps to a more effective management of chronic HBV infection and moves the aim of HBV eradication closer to realization (Yu et al.). The study conducted by Schlesinger et al., regarding the countries management of Dengue virus outbreaks demonstrated that Migration and/or socioeconomic status are factors that might impact predictive performance and should be further evaluated. Overall early warning and response system (EWARS) performed very well, providing evidence that it should continue to be implemented in countries for outbreak prediction (Schlesinger et al.).

Finally, the concept of a serious global pandemic named “Disease X” was raised and discussed in this Research Topic. Indeed, “Disease X” refers to an unexpected and unknown outbreak of a contagious or infectious disease caused by a “Pathogen X,” which is presently unidentified and capable of infecting humans. This pathogen X is most likely a zoonotic virus (4, 5). According to the World Health Organization (WHO), the ”Disease X“ is considered among highly contagious diseases such as Ebola, Zika, and COVID-19 (Zaman et al.). For Scientists, as pathogen X is an elusive pathogen, we are unable to prevent the occurrence of Disease X. However, by implementing preventative measures, we may be able to impede or minimize its transmission and possible health risks. In order to achieve this, a universal scientific protocol for managing and controlling “Disease X” would be required.
